# Modelling effective diffusion for accurate NMR pore size analysis in nano- and microporous rocks

**DOI:** 10.1038/s41598-025-20379-x

**Published:** 2025-10-21

**Authors:** Michał Fajt, Grzegorz Machowski, Bartosz Puzio, Artur T. Krzyżak

**Affiliations:** Faculty of Geology, Geophysics and Environmental Protection, AGH University of Krakow, al. Adama Mickiewicza 30, Kraków, 30-059 Poland

**Keywords:** Low-field NMR (LF-NMR), Pore size distribution (PSD), Diffusion, Internal gradients, Surface relaxivity, Chemistry, Materials science, Solid Earth sciences

## Abstract

Low-field NMR (LF-NMR) is a widely applied technique for evaluating pore size distribution (PSD) in porous materials. Conventional approaches typically assume surface-controlled spin-spin relaxation and negligible diffusion contributions under the fast-diffusion regime, which introduces systematic errors when applied to nano- and microporous systems. In this work, we present the Effective Diffusion Cubic (EDC) model, a new framework for LF-NMR-based PSD estimation in tight rocks. The EDC method incorporates pore-size dependence of both the effective diffusion coefficient and the induced internal magnetic field gradient. Crucially, the effective diffusion coefficient, *D(d)*, is parameterized by a logistic function that faithfully approximates the Padé form, enabling a precise quantification of diffusion-related effects on T_2_ relaxation. Applied to nine siliciclastic core samples, the EDC approach produced PSDs corrected for diffusion-induced distortions and in closer agreement with independent reference data compared to conventional models. These results demonstrate that the EDC methodology provides a physically consistent and more accurate means of quantifying pore systems, thereby enhancing NMR-based petrophysical characterization of tight rock formations.

**Supplementary Information:**

The online version contains supplementary material available at 10.1038/s41598-025-20379-x.

## Introduction

Pore size distribution (PSD) is a fundamental property of porous rocks that governs fluid storage and transport, allowing an assessment of their potential as hydrocarbon or geothermal reservoirs^1,2^. Traditional PSD measurement methods, such as mercury injection capillary porosimetry (MICP) and low-temperature nitrogen adsorption (LTNA), often suffer from destructive sample preparation, PSD detection limits, and poor resolution in their proximity^3,4^. Therefore, due to its non-destructive and non-invasive nature, as well as sensitivity to all pore size ranges, low-field nuclear magnetic resonance (LF-NMR) has emerged as a powerful tool in geophysical prospecting, particularly for the exploration of reservoir rocks and their pore space characterization^2,5–8^. However, in nano- and microporous siliciclastic systems such as tight sandstone and shale formations, accurate PSD quantification is particularly challenging due to limited pore accessibility, high clay mineral content, and low porosity. Most of these barriers can be mitigated, e.g., by using differential LF-NMR distributions, devoid of information from^1^H protons bound in the rock-matrix lattice, associated with organic matter, clay matter, and closed porosity^9,10^. Nevertheless, difficulties related to the presence of internal gradients (*G*), as well as diffusion (*D*) restrictions in rocks with high magnetic susceptibility and nano- and micrometric rock pore space, are still hard to overcome^9,11–18^.

Standard LF-NMR PSD estimation methods assume a fast diffusion regime, also referred to as the short-time (ST) regime, where the diffusion coefficient is constant and NMR transverse relaxation time (*T*_*2*_) is dominated solely by surface relaxation (*T*_*2S*_)^19^. This simplification yields a direct, linear relationship between T_2_ relaxation rate and surface-to-volume ratio (*S/V*) and allows a straightforward T_2_-PSD transformation using constant surface relaxivity, *ρ*_*2*_^5,20^.

However, in nano- and microporous systems, since pore dimensions become smaller than the diffusion path of observed ¹H nuclei, the system transitions into the motionally averaging (MAV) or long-time diffusion regime. In this regime, diffusion is no longer fast; instead, it becomes restricted, and, as a result, the apparent diffusion coefficient decreases^21,22^. Additionally, the extent of the influence of internal gradients on signal decay is inversely proportional to pore size and can cause substantial distortions in nano- and micro-pore space even when applying low field (0.05 T) and Carr–Purcell–Meiboom–Gill (CPMG) pulse sequence with low echo times^9^. As a result, this increases the contribution of the diffusion component (*T*_*2D*_) in nano- and micropores to overall T_2_ relaxation. Some authors have also pointed out that the experimentally derived apparent diffusion coefficient in nano- and micropore space is smaller than would result from changes to the diffusion regime alone. Many studies have highlighted correlations between magnetic susceptibility and surface relaxivity in siliciclastic rocks, Refs.^17,18^ as well as the role of paramagnetic mineral content in modifying observed T₂ relaxation times^9,13–16^. However, there is only a limited discussion in the literature regarding the simultaneous impact of diffusion regimes and internal gradients on T₂ relaxation measurements in porous rock systems^14,23–25^.

In the field of LF-NMR-based PSD estimation, numerous studies and methods have been developed to date. When linear T₂-to-PSD conversion proves inadequate, approaches based on power-law relationships are commonly applied. These methods, however, typically neglect the influence of diffusion on the calculated surface relaxivity^4,9,26,27^. Several studies have attempted to incorporate a diffusion component into pore size distribution estimation by utilising D-T_2_ maps to determine surface relaxivity, which is then used for T_2_-PSD conversion. However, such approaches have typically focused on systems with high surface relaxivity values^28^ or carbonate rocks with large, well-connected pores^29^ and have largely neglected the influence of internal magnetic field gradients induced by susceptibility contrasts. Recent studies on chert rocks introduced T₂-based PSD conversions incorporating the effects of induced magnetic field gradients. Yet, the effective diffusion functions used in these approaches were based on literature-derived values for water diffusion in pure silica nanopores < 4 nm, limiting their applicability in mineralogically diverse rocks or when fluids other than water are used in LF-NMR experiments^11,30^. Other authors have proposed methods using dual surface relaxivity values, assigning different *ρ*_*2*_ values to macropore and micropore regions, acknowledging that parts of the T_2_ distribution deviate from a linear pore size correlation^31,32^. Nevertheless, despite these advances, no consensus has yet been reached on a universal method for converting LF-NMR to PSD in tight pore space that simultaneously mitigates all challenges.

Conventional models, defined here for comparative purposes as the Negligible Diffusion Linear (NDL) and Free Diffusion Quadratic (FDQ), rely on the assumptions of surface relaxation dominance and a fast-diffusion regime across the entire pore-size spectrum. While computationally convenient, such assumptions are insufficient to capture the true relaxation dynamics in nano- and microporous systems, where diffusion restriction and internal magnetic field gradients strongly influence the spin-spin relaxation process.

To overcome these limitations, we propose the Effective Diffusion Cubic (EDC) framework, designed to derive absolute pore size distributions from LF-NMR data. Within EDC, the pore-size-dependent internal gradient, *G(d)*, is explicitly defined, while the effective diffusion coefficient, *D(d)*, is represented by a logistic function that accurately reproduces the Padé approximation of the diffusion behavior in confined geometries. This functional form enables a rigorous incorporation of diffusion-related contributions into the relaxation model, providing a more realistic mapping of T2 distributions into PSDs. Furthermore, structural parameters of *D(d)* are determined through simulations and integrated with percolation theory, allowing differential LF-NMR responses to be transformed into physically consistent pore size distributions. Using this methodology, we assess the robustness of the EDC approach across a representative set of siliciclastic rocks and benchmark its performance against the NDL and FDQ models.

## Materials and methods

### Samples characterization

The tested materials comprised 9 siliciclastic rock-core samples: 3 sandstones (S1-3), 3 mudstones (M1-3), and 3 heteroliths (H1-3) of Miocene age. Samples were taken from 8 wells located in southeastern Poland, in the Carpathian Foredeep area, targeting the geological strata of the autochthonous Miocene formations (Supplementary Material Figure S1).

For the purposes of the study, nine pairs of cylindrical core samples were extracted from the drill cores at corresponding depths in the range of 333.5–2512.4 m b.g.l. Each extracted core had a diameter of approximately 2.2–2.5 cm and a length of 2–5 cm. One specimen from each pair was destined for LF-NMR measurements. The corresponding cores were subsequently crushed and divided into three sub-samples, which were allocated to MICP, LTNA, and magnetic susceptibility analyses, respectively.

### Mercury injection capillary pressure (MICP)

MICP measurements were performed using an Auto Pore IV 9520 (Micromeritics, USA) mercury porosimeter. Before the analysis, all samples were crushed and dried at 105 °C for 24 h to remove moisture from the pore space. The pressure was measured at 120 points in the 0.003–414 MPa range, which corresponds to pore sizes from about 500 μm to 3–4 nm. Equivalent pore throat diameters were computed based on the corresponding capillary pressure using the Washburn equation^33^:

1 $$\:{d}_{MICP}=-\frac{4{\gamma\:}_{Hg}{cos\theta\:}_{Hg}}{{P}_{c}}$$

where: *d*_*MICP*_ is pore size (m); *γ*_*Hg*_ air/mercury surface tension (*γ*_*Hg*_ *= 0.485* N/m); *θ*_*Hg*_ is the contact angle between mercury and a porous material (*θ*_*Hg*_ *= 130°*) and *P*_*c*_ is capillary pressure (Pa).

The value of the permeability coefficient was determined by the Katz-Thomson method, in which this coefficient is calculated according to the following formula^34^:

2 $$\:K=\:\frac{1}{89}\cdot{L}_{max}^{2}\cdot\frac{{L}_{max}}{{L}_{char}}\cdot\varphi\:{S}_{Lmax}$$

where: *K* is the absolute permeability coefficient (m^2^); *L*_*max*_ is the maximum length at which the highest hydraulic conductivity occurs (m); *L*_*char*_ is the characteristic pore length (m), determined based on the threshold pressure *P*_*th*_ and Washburn’s equation^35,36^; *ϕ* is effective porosity (m^3^/m^3^) and *S*_*Lmax*_ is the volume of effective pores with a width greater than or equal to *L*_*max*_ (m^3^).

### Low-temperature nitrogen adsorption (LTNA)

The characterization of the porous texture was carried out based on low-temperature nitrogen adsorption-desorption isotherms at − 196 °C. The measurements were performed using a high-precision NOVA 800 surface area and porosity analyser (Anton Paar) over a wide range of relative pressures, *P*/*P*_*0*_ from approximately 1 × 10⁻³ to 0.99. Before analysis, the crushed rock samples were dried under a vacuum at 200 °C for 12 h. For each sample, the specific surface area was calculated using the Brunauer–Emmett–Teller (BET) method^37^. Total pore volume was estimated at a *P/P*_*0*_ *= 0.99*. Mesopore volume, corresponding to pores with diameters between 2 and 50 nm, was calculated using the NLDFT adsorption model implemented in the Kaomi 2.0 software (Anton Paar) for the NOVA instrument.

### Magnetic susceptibility

Magnetic susceptibility (*χ*_*Sample*_) was determined using a Bartington MS2 meter (Bartington Instruments Ltd.), equipped with a sensor for laboratory measurements. The sensor employed consists of a thermally stable oscillator paired with an induction coil, where the coil’s influence modulates the oscillator’s frequency. In the absence of any magnetically active materials, the oscillator frequency reflects only the magnetic permeability of the surrounding air. However, when a magnetically susceptible sample is placed near the coil, its presence alters the local magnetic permeability, thereby shifting the oscillator’s frequency. The instrument then automatically calibrates this frequency shift and translates it into a magnetic susceptibility value.

### Low-field nuclear magnetic resonance (LF-NMR) experiments

1D-T_2_ LF-NMR experiments were conducted using a 2 MHz Rock Core Analyser by Magritek. Samples were prepared in both dried and kerosene-saturated states. This was achieved through drying at 110 °C and kerosene saturation at vacuum conditions of 0.007 bar for 12 h, respectively. Measurements were carried out applying T_2_ Carr − Purcell − Meiboom − Gill (CPMG) with residual time, *RT = 5000* ms; number of echoes, *NoE = 50,000*; echo time, *TE = 60* us, and number of scans, *NoS = 512*. The distributions of T_2_ relaxation times were derived using the Inverted Laplace Transform (ILT). To avoid additional bias in the relative comparison of pore size distributions across the heterogeneous dataset, we fixed the smoothing factor at *α = 1* for all samples. This value was located within the smallest chi-squared statistic region in every case, ensuring the stable and comparable ILT solutions. Additionally, differential distributions were calculated by the subtraction of background, rock matrix, clay minerals, organic matter, and closed pores T_2_ spin-echo signal (dry state sample signal) from the signal of the 100% kerosene-saturated sample before ILT. These distributions were later used in the LF-NMR PSD estimation and conversion framework setting, as they represent only the effective porosity accessible for both reference methods, MICP and LTNA^9,10^.

### LF-NMR effective diffusion simulations

In the short-time (ST) diffusion limit, diffusive behaviour is essentially the same as the free diffusion regime, and the apparent diffusion coefficient, *D*_*ST*_, approaches a constant value of *D*_*0*_. This behaviour is precisely described by Mitra’s relation expressed by equation^38^:

3 $$\:\frac{{D}_{ST}}{{D}_{0}}=1-\frac{4\sqrt{{D}_{0}t}}{9\sqrt{\pi\:}}\cdot\:{\left(\frac{S}{V}\right)}_{pore}$$

Using a CPMG sequence with constant echo time, with decreasing pore size, the fluid molecules will diffuse, probing more and more interconnected pore spaces in the same diffusion time, thus entering the long-time (motionally averaging, MAV) limit, where the apparent diffusion coefficient, *D*_*MAV*_, approaches a constant value of $\:{D}_{\infty\:}$, as expressed by equation^39^:

4 $$\:\frac{{D}_{MAV}}{{D}_{0}}=\frac{{D}_{\infty\:}}{{D}_{0}}+\frac{{\beta\:}_{1}}{t}+\frac{{\beta\:}_{2}}{{t}^{3/2}}$$

where: *τ* is the tortuosity coefficient (-), which mathematically can be described as the ratio of length over tortuous paths to the length of the sample; $\:{\beta\:}_{1}=\frac{\left(1-\phi\:\right)d}{8{D}_{0}}$ and $\:{\beta\:}_{2}=\frac{1-\phi\:}{4\sqrt{\pi\:}}\cdot\:{\left(\frac{d}{2{D}_{0}}\right)}^{3/2}$ are constants dependent on rock pore space characteristics^40^.

By using Eqs. 3 and 4, we can obtain apparent diffusion coefficients ​in the limits of short-time (*D*_*ST*_) and long-time diffusion (*D*_*MAV*_), respectively. In cases where these two limits are known, a two-point Padé approximation can be used to interpolate between them to further obtain an extension of *D*_*Padé*_ for the intermediate-time diffusion. Therefore, introducing $\:B=\frac{4\sqrt{{D}_{0}}\:}{9\sqrt{\pi\:}}\cdot\:S/V$ we can obtain^41^:

5 $$\:\frac{{D}_{\text{P}\text{a}\text{d}\text{e}}}{{D}_{0}}=1-\left(1-\frac{{D}_{\infty\:}}{{D}_{0}}\:\right)\cdot\:\frac{B\sqrt{t}+\left(1-\frac{{D}_{\infty\:}}{{D}_{0}}\right)\cdot\:\frac{t}{\theta\:}}{\left(1-\frac{{D}_{\infty\:}}{{D}_{0}}\right)+B\sqrt{t}+\left(1-\frac{{D}_{\infty\:}}{{D}_{0}}\:\right)\cdot\:\frac{t}{\theta\:}}$$

where: *θ* is the structural parameter with time dimension (s). In an ideal spherical pore system, $\:\sqrt{{D}_{0}\theta\:}$ is expected to be proportional to pore diameter, *d*, with a proportionality factor, which can be fitted during two-point Padé approximation^41^.

The direct application of the Padé approximation (Eq. 5) to the PSD estimation workflow would be impractical to use because of the significant number of input variables. Therefore, in this study, we used a modified form of the logistic function as an alternative approximation for the apparent diffusion coefficient, *D*_*Log*_, expressed as:

6 $$\:\frac{{D}_{Log}}{{D}_{0}}=\frac{\left(\frac{{D}_{\infty\:}}{{D}_{0}}-1\right){d}_{c}}{{d}_{c}+d}+1$$

where: *d*_*c*_ is the characteristic pore size (m) corresponding to the centre of the sigmoid.

To investigate the relationship between effective diffusion behavior and pore size in nano- to microscale domains, we performed numerical simulations for artificial, spherical pores with broad size ranges (0.1 nm ≤ d ≤ 0.1 mm) and various levels of diffusion restriction (8.8⸱10^− 13^ m^2^/s ≤ D_∞_ ≤ 8.8⸱10^− 10^ m^2^/s). Using Eqs. 3 and 4, effective diffusion coefficients in short (D(d)_ST_) and long-time (D(d)_MAV_) diffusion limits were obtained. Then, two-point approximations were performed, solving for the proportionality factor of the structural parameter, *θ*, to obtain effective diffusion coefficients, *D(d)*_*Padé*_, in the full range of considered pore sizes. Finally, logistic functions, *D(d)*_*Log*_, could be estimated by solving for characteristic pore size, *dc*, using mean absolute error in logarithmic space (logMAE_D(d)_) between *D(d)*_*Padé*_ and *D(d)*_*Log*_ effective diffusion coefficients minimisation as described:

7 $$\:{\text{logMAE}}_{D\left(d\right)}=\frac{\sum\:_{i=1}^{n}\left|log\left({D\left(d\right)}_{Log\left(i\right)}\right)-log\left({D\left(d\right)}_{Pade\left(i\right)}\right)\right|}{n}$$

where: *D(d)*_*Log(i)*_ is the effective diffusion coefficient at each *i-th* pore size derived from Eq. 6 (m^2^/s); *D(d)*_*Padé(i)*_ is the effective diffusion coefficient at each *i-th* pore size derived from Eq. 5 (m^2^/s), and n is the number of points.

### LF-NMR PSD estimation: negligible diffusion linear (NDL) model

For fluid contained within a porous medium, the transverse relaxation time can be divided into three components, known as bulk relaxation (*T*_*2B*_), surface relaxation (*T*_*2S*_), and diffusion component (*T*_*2D*_), which can be expressed as^19^:

8 $$\:\frac{1}{{T}_{2}}=\frac{1}{{T}_{2B}}+\frac{1}{{T}_{2S}}+\frac{1}{{T}_{2D}}$$

In the fast diffusion regime, using short echo times in a low magnetic field, it can be assumed that diffusion is instantaneous for samples with low magnetic field gradients, and its effects on relaxation will be negligible during the T_2_ experiment. Therefore, by neglecting the diffusion component, we obtain:

9 $$\:\frac{1}{{T}_{2}}\approx\:\frac{1}{{T}_{2B}}+\frac{1}{{T}_{2S}}\approx\:\frac{1}{{T}_{2B}}+{\rho\:}_{2}\cdot\:\frac{2Fs}{d}$$

where: *ρ*_*2*_ is transverse surface relaxivity (m/s) and *F*_*s*_ is the pore shape factor, which for spherical, cylindrical, and planar geometries is equal to 3, 2 and 1, respectively (-) and *d* is the pore diameter (m). For the T_2_-PSD conversion, we adopted a spherical pore geometry, *Fs = 3*, consistent with the assumption used in the effective diffusion simulations.

Then introducing $\:C=\frac{1}{{T}_{2}}-\frac{1}{{T}_{2B}}$ the pore size, *d*, can then be calculated using the linear equation:

10 $$\:{d}_{lin}={\rho\:}_{2}\cdot\:\frac{2Fs}{C}$$

### LF-NMR PSD estimation: free diffusion quadratic (FDQ) model

The extent of the magnetic field gradient results primarily from the differences in magnetic susceptibility between the pore-filling fluid and the rock matrix of the core sample. Because the relationship between magnetic field gradients and diffusive relaxation is quadratic for rocks with a noticeable amount of paramagnetic minerals and particularly small pores, the diffusive relaxation component in Eq. 8 cannot be neglected^11,30^.

In isotropic, siliciclastic rocks, it can be assumed that paramagnetic minerals are evenly dispersed within the granular rock matrix of the sample. Therefore, the magnetic field gradient, *G*, should be uniform in the whole sample volume, thus, for pores of size *d*, its extent can be expressed by:

11 $$\:G\left(d\right)\approx\:\frac{\left({\chi\:}_{fluid}-{\chi\:}_{sample}\right)\cdot\:{B}_{0}}{d}=\frac{\varDelta\:\chi\:{B}_{0}}{d}$$

where: *χ*_*fluid*_ is the volume magnetic susceptibility of pore-filling fluid (-); *χ*_*sample*_ is the volume magnetic susceptibility of rock core matrix (-); *∆χ* is the volume magnetic susceptibility gradient (-) and *B*_*0*_ is the magnetic field induction (T).

Therefore, considering the free diffusion regime, where the apparent diffusion coefficient, *D*, approaches a constant value of *D*_*0*,_ and substituting Eq. 11 into Eq. 8, we can obtain:

12 $$\:\frac{1}{{T}_{2}}=\frac{1}{{T}_{2B}}+\frac{1}{{T}_{2S}}+\frac{1}{{T}_{2D}}\approx\:\frac{1}{{T}_{2B}}+{\rho\:}_{2}\cdot\:\frac{2Fs}{d}+\frac{{D}_{0}\cdot\:{\left(\gamma\:G\left(d\right){t}_{e}\right)}^{2}}{12}$$

Then by using constant *C* and introducing $\:F=\frac{{\left(\gamma\:\varDelta\:\chi\:{B}_{0}{t}_{e}\right)}^{2}}{12}$, Eq. 12 can be further expressed as:

13 $$\:C-{\rho\:}_{2}\cdot\:\frac{2Fs}{d}-\frac{{D}_{0}F}{{d}^{2}}=0$$

which can be reduced into a general quadratic function form:

14 $$\:C{d}^{2}-2Fs{\rho\:}_{2}d-{D}_{0}F=0$$

with two solutions, *j = 1*,*2*, of which only one will be true:

15 $$\:{d}_{quad,j}=\frac{2Fs{\rho\:}_{2}\pm\:\sqrt{{\left({2Fs\rho\:}_{2}\right)}^{2}+4CF{D}_{0}}}{2C}$$

### LF-NMR PSD estimation: effective diffusion cubic (EDC) model

As described earlier, with decreasing pore size, the fluid molecules will diffuse, probing more and more interconnected pore spaces in the same diffusion time. Thus, the assumption of the free diffusion regime applied in conventional approaches is no longer valid. Molecules diffusing in such a system begin to encounter restrictions imposed by the pore walls. For this reason, the diffusion coefficient in Eq. 8 cannot be approximated as a constant, *D*_*0*_ value, which requires an appropriate correction. Thus, considering the results of simulations, diffusion in porous media can be described as the effective change in the apparent diffusion coefficient depending on the pore size *d*. Subsequently, assuming *D = D(d)* and substituting Eq. 11 into Eq. 8, we can obtain:

16 $$\:\frac{1}{{T}_{2}}=\frac{1}{{T}_{2B}}+\frac{1}{{T}_{2S}}+\frac{1}{{T}_{2D}}\approx\:\frac{1}{{T}_{2B}}+{\rho\:}_{2}\cdot\:\frac{2Fs}{d}+\frac{D\left(d\right)\cdot\:{\left(\gamma\:G\left(d\right){t}_{e}\right)}^{2}}{12}$$

Then we can further reduce this expression by using introduced constants *C*, *F*, and substituting the logistic approximation of the *D(d)* function described in Eq. 6 to Eq. 16:

17 $$\:C-{\rho\:}_{2}\cdot\:\frac{2Fs}{d}-\frac{\left({D}_{\infty\:}{-D}_{0}\right)F{d}_{c}}{\left({d}_{c}+d\right){d}^{2}}-\frac{{D}_{0}F}{{d}^{2}}=0$$

which can be transformed to the general form of the cubic function:

18 $$\:{d}^{3}+\frac{\left(C{d}_{c}-{2\rho\:}_{2}Fs\right)}{C}{d}^{2}-\frac{({2\rho\:}_{2}Fs{d}_{c}+{D}_{0}F)}{C}d-\frac{{D}_{\infty\:}F{d}_{c}}{C}=0$$

19 $$\:{d}^{3}+a{d}^{2}+bd+c=0$$

$$\:\text{w}\text{h}\text{e}\text{r}\text{e}:\:a=\frac{\left(C{d}_{c}-{2\rho\:}_{2}Fs\right)}{C};\:b=-\frac{({2\rho\:}_{2}Fs{d}_{c}+{D}_{0}F)}{C}\:\text{a}\text{n}\text{d}\:c=-\frac{{D}_{\infty\:}F{d}_{c}}{C}.$$.

Subsequently, using the Cardano equations, i.e., substituting $\:d=x-\frac{a}{3}$ and introducing $\:p=b-\frac{{a}^{2}}{3}$ and $\:q=c-\frac{ab}{3}+\frac{2{a}^{2}}{27}$ the quadratic expression from Eq. 19 can be eliminated, thus we obtain:

20 $$\:{x}^{3}+px+q=0$$

with one rational, *j = 1* and two complex, *j = 2*,*3* solutions of which only one will be true:

21 $$\:{d}_{cub,1}=\:\sqrt[3]{-\frac{q}{2}+\sqrt{\varDelta\:}}+\sqrt[3]{-\frac{q}{2}-\sqrt{\varDelta\:}}-\frac{a}{3}$$

22 $$\:{d}_{cub,2}=\:\omega\:\cdot\:\sqrt[3]{-\frac{q}{2}+\sqrt{\varDelta\:}}+{\omega\:}^{2}\cdot\:\:\sqrt[3]{-\frac{q}{2}-\sqrt{\varDelta\:}}-\frac{a}{3}$$

23 $$\:{d}_{cub,3}={\omega\:}^{2}\cdot\:\:\sqrt[3]{-\frac{q}{2}+\sqrt{\varDelta\:}}+\omega\:\cdot\:\sqrt[3]{-\frac{q}{2}-\sqrt{\varDelta\:}}-\frac{a}{3}$$

$$\:\text{w}\text{h}\text{e}\text{r}\text{e}:\:\varDelta\:\:={\left(\frac{q}{2}\right)}^{2}+{\left(\frac{p}{3}\right)}^{3}\:\text{a}\text{n}\text{d}\:\omega\:=-\frac{1}{2}+i\frac{\sqrt{3}}{2}.$$

### LF-NMR T_2_-PSD conversion framework

The overall approach for converting LF-NMR experimental data into a pore size distribution (PSD) is illustrated in Supplementary Material Figure S2. To obtain a reference full-range pore size distribution (PSD) for each rock sample, we combined the results from LTNA and MICP. Each method probes a distinct range of pore sizes: LTNA measurements are sensitive to nano- and micropores (typically 1.7–200 nm), whereas MICP primarily detects micro-, meso-, and macropores (> 10 nm). To construct a combined curve of incremental PSDs, we identified the pore size range in which both methods exhibited overlapping measurement sensitivity. Within this transitional region, we selected a connection point based on the visual inspection of the amplitude convergence between the two distributions^42^. In all cases, the chosen connection point was located within the range of 5–10 nm, where the incremental frequencies of LTNA and MICP data intersected or closely matched (Fig. 2). The combined PSD curve was then generated by retaining the LTNA-derived distributions below the connection point and merging them with the MICP-derived distributions above this threshold.

To define the conversion points, an approach based on the percolation threshold was employed, as proposed in previous works^9,43,44^. According to percolation theory, for a given pore diameter d, the cumulative MICP + LTNA curve represents the accessible pore fraction Xᴬ(d), while the inverse cumulative LF-NMR T₂ curve corresponds to the bond occupation probability X(d). Relying on the simulation findings, the following relationship is used^45^:

24 $$\:X\left(d\right)={X}^{A}\left(d\right)\:for\:\text{Z}{X}^{A}\left(\text{d}\right)\:>\:2.5$$

where *Z* denotes the coordination number, which is derived from the percolation threshold, *p*_*c*_, using the empirical expression^46^:

25 $$\:{p}_{c}=0.627{e}^{-0.144Z}$$

The percolation threshold, *p*_*c*_, is defined as the point of maximum slope of the MICP curve, where mercury first enters a continuous, connected pore network – effectively forming the initial sample-spanning cluster. Once this threshold is identified, the accessible fraction *ZX*^A^(*d*) could be computed, thus conversion points could be identified based on Eq. 24.

Following this step, the LF-NMR PSD could be estimated by solving *ρ*_*2*_ in the case of the NDL and FDQ model and *ρ*_*2*_ and *D*_*∞*_ in the case of the EDC model, assuming spherical pore geometry to estimate the pore diameters, *d*_*NMR(k)*_ at each relaxation time, *T*_*2(k)*_ from the differential LF-NMR distribution satisfying assumption described in Eq. 24. The optimal values of restricted diffusion coefficient, *D*_*∞*_ were obtained accounting for the physical lower limit of detectable pore sizes (d_min_ > 0.6 nm), constrained by the dynamic molecular size of kerosene particle by using Eqs. 27, 28 for characteristic pore sizes, d_c_ functions estimated during effective diffusion, *D(d)* simulations. Simultaneously, the surface relaxivity, *ρ*_*2*_, was determined by minimising the porosity-weighted mean absolute error (logMAE_PSD_) in logarithmic space between the pore sizes derived from combined MICP + LTNA data and those estimated from LF-NMR, as defined below:

26 $$\:{\text{logMAE}}_{{PSD}}=\frac{\sum\:_{i=1}^{n}{f}_{NMR\left(k\right)}\cdot\:\left|\text{l}\text{o}\text{g}\left({d}_{NMR\left(k\right)}\right)-{\text{l}\text{o}\text{g}(d}_{MICP+LTNA\left(k\right)})\right|}{n}$$

where: *f*_*NMR(k)*_ represents the fraction of normalised porosity detected at each *T*_*2(k)*_; *d*_*NMR(k)*_ is the pore size estimated using NDL (*d*_*lin*_), FDQ (*d*_*quad(j)*_) or EDC (*d*_*cub(j)*_) model from each *T*_*2(k)*_ (m) at *X(d)*; *d*_*MICP+LTNA(k)*_ is the pore size derived by combining LTNA and MICP distributions (m) at each corresponding *ZXA(d)*, and n is the number of conversion points.

Once the conversion coefficients were calibrated, the T₂ relaxation time distributions could be transformed into absolute PSDs using Eqs. 10, 15, and 21–23 for the NDL, FDQ, and RDC models, respectively. However, using Eq. 15, it was impossible to determine a reliable surface relaxivity for most samples. Consequently, PSDs obtained by the FDQ method were calculated using the EDC *ρ*_*2*_ values (Table 1).

## Results

### Simulation of effective diffusion in spherical pore space

**Fig. 1:**
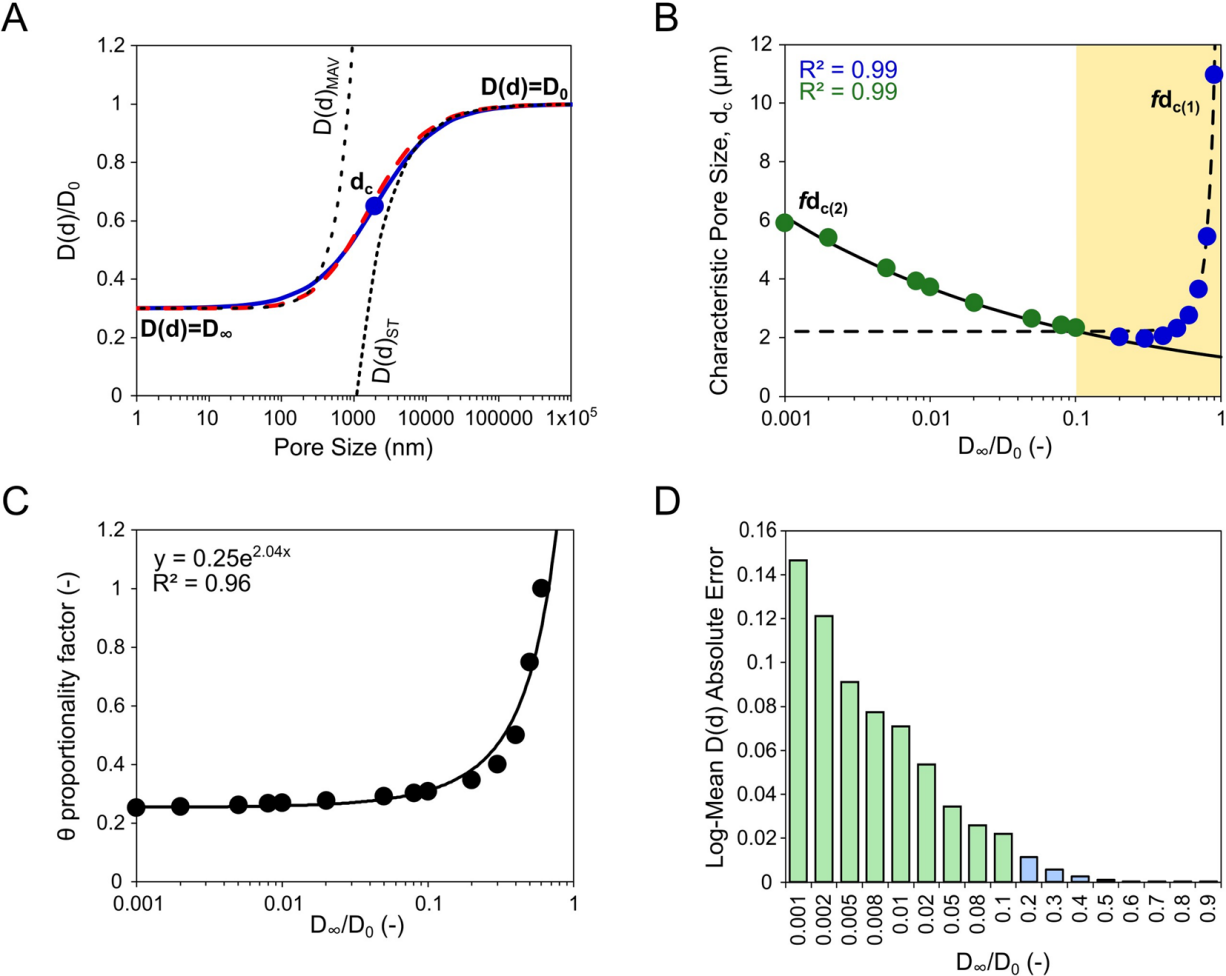
Results of effective diffusion functions, *D(d)* simulations for spherical pore systems. **(A)** Normalised effective diffusion functions, *D(d)* simulated for *D*_*∞*_*/D*_*0*_ *= 0.2* according to Padé, *D(d)*_*Padé*_ (red, dashed curve) and logistic, *D(d)*_*Log*_ (blue curve) approximation with indication of short-time, *D(d)*_*ST*_ and long-time, *D(d)*_*MAV*_ diffusion functions and obtained characteristic pore size, *d*_*c*_ point; **(B)** Results of the characteristic pore size, d_c_ points fitting obtained during the D(d)_Log_ simulations approximated by exponential, *fd*_*c(1)*_ (blue points) and power, *fd*_*c(2)*_ (green points) functions. The yellow area indicates *D*_*∞*_*/D*_*0*_ range of the *fd*_*c(1)*_ function; **(C)** Results of the proportionality factor fitting for structural parameter, *θ*, during the *D(d)*_*Padé*_ simulations with fitted exponential function. **(D)** Log**-**mean absolute fitting errors between the Padé and the obtained logistic approximation of effective diffusion coefficients, *logMAE*_*D(d)*_, obtained using *fd*_*c(1)*_ (blue bars) and *fd*_*c(2)*_ (green bars) functions for assumed *D*_*∞*_.

To investigate the relationship between effective diffusion and pore size in nano- to microscale domains, we performed numerical simulations of the effective diffusion functions, *D(d)* for artificial, spherical pores with broad size ranges and different magnitudes of restricted diffusion coefficients, *D*_*∞*_ (Fig. 1A). As a result of the structural parameters fitting, it was possible to determine the dual nature of the logistic approximation structural parameter – characteristic pore size, *d*_*c*_ and *D*_*∞*_ relationship (Fig. 1B) which could be described using the equations:

27 $$\:{fd}_{c\left(1\right)}=2.22+{0.00154\cdot\:e}^{\left({9.60\cdot\:D}_{\infty\:}/{D}_{0}\right)}\:for\:{D}_{\infty\:}/{D}_{0}>0.1$$

28 $$\:{fd}_{c\left(2\right)}={1.34\left({D}_{\infty\:}/{D}_{0}\right)}^{-0.22}\:for\:{D}_{\infty\:}/{D}_{0}<0.1$$

At the same time, the proportionality factor of the Padé approximation structural parameter, *θ*, did not show the dual nature of the correlation with *D*_*∞*_ and could be approximated by a single exponential function (Fig. 1C). Additionally, the registered effect of diffusion restriction on obtained values in the range of *D*_*∞*_*/D*_*0*_ *< 0.1* is almost negligible. The proposed logistic form of approximation, *D(d)*_*Log*_ between short-time, *D(d)*_*ST*_ and long-time, *D(d)*_*MAV*_ diffusion functions, reconstructed the more complicated form of Padé approximation, *D(d)*_*Padé*_ with minor fitting errors, especially in less restricted pore systems, with *D*_*∞*_*/D*_*0*_ *> 0.1*. However, the fitting error constantly increased with decreasing magnitude of restricted diffusion coefficient, D_∞_. Nevertheless, at its maximum log-mean absolute fitting error (*logMAE*_*D(d)*_), effective diffusion approximated by *D(d)*_*Log*_ did not exceed the *D(d)*_*Padé*_ model by more than 1.4 times (Fig. 1D).

### Validation of T₂-PSD conversion results using introduced, EDC, and conventional models

**Fig. 2:**
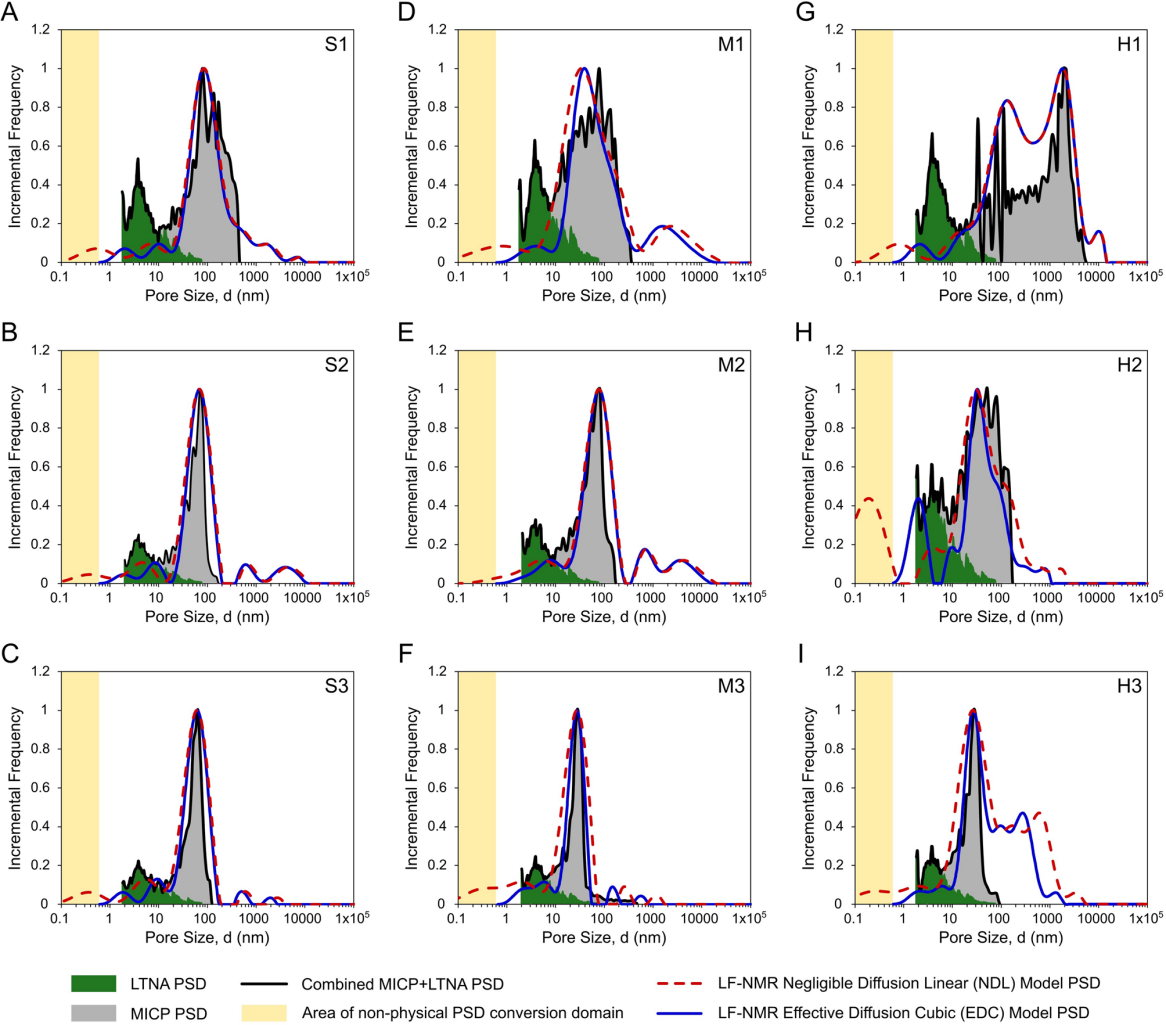
Results of EDC and NDL models LF-NMR PSD estimation against reference methods data across representative rock-core samples. Comparison of pore size distributions (PSDs) obtained from reference low-temperature nitrogen adsorption (LTNA, green areas), mercury injection capillary porosimetry (MICP, grey areas) methods, and LF-NMR using the Negligible Diffusion Linear (NDL, red, dashed lines) and Effective Diffusion Cubic (EDC, blue lines) models for nine studied samples: **(A–C)** sandstones (S1-S3); **(D–F)** mudstones (M1-M3) and **(G–I)** heteroliths (H1–H3). Yellow areas indicate the non-physical pore sizes conversion domain located beyond the lower limit of the dynamic particle diameter of kerosene (*d* < 0.6 nm). Black lines represent combined MICP + LTNA PSD distributions used in the estimation of conversion parameters for NDL and EDC LF-NMR PSD models.

Initially, we compared EDC outcomes against those obtained via the conventional NDL method and benchmarked them against reference methods, namely the combined MICP and LTNA PSD distributions for nine measured rock-core samples (Fig. 2). In nearly all analysed cases (Fig. 2A–I), the NDL T₂-PSD conversion approach significantly underestimated pore sizes in the low-end range, extending into the non-physical pore sizes domain, which lies beyond the dynamic particle diameter of the working fluid used, namely kerosene. This artefact was particularly prominent in samples with high nano- and microporosity (Fig. 2F, H-I).

By contrast, the proposed EDC model demonstrated markedly improved alignment with the combined LTNA and MICP distributions (black lines). Notably, the EDC approach prevented the low-end artefacts and captured pore size distributions with greater accuracy, providing a closer match to experimentally measured PSD profiles. This improvement was particularly evident in samples exhibiting a mode in the nano-to-micropore range of *d* < 100 nm (Fig. 2B-C, F, H-I).

**Fig. 3:**
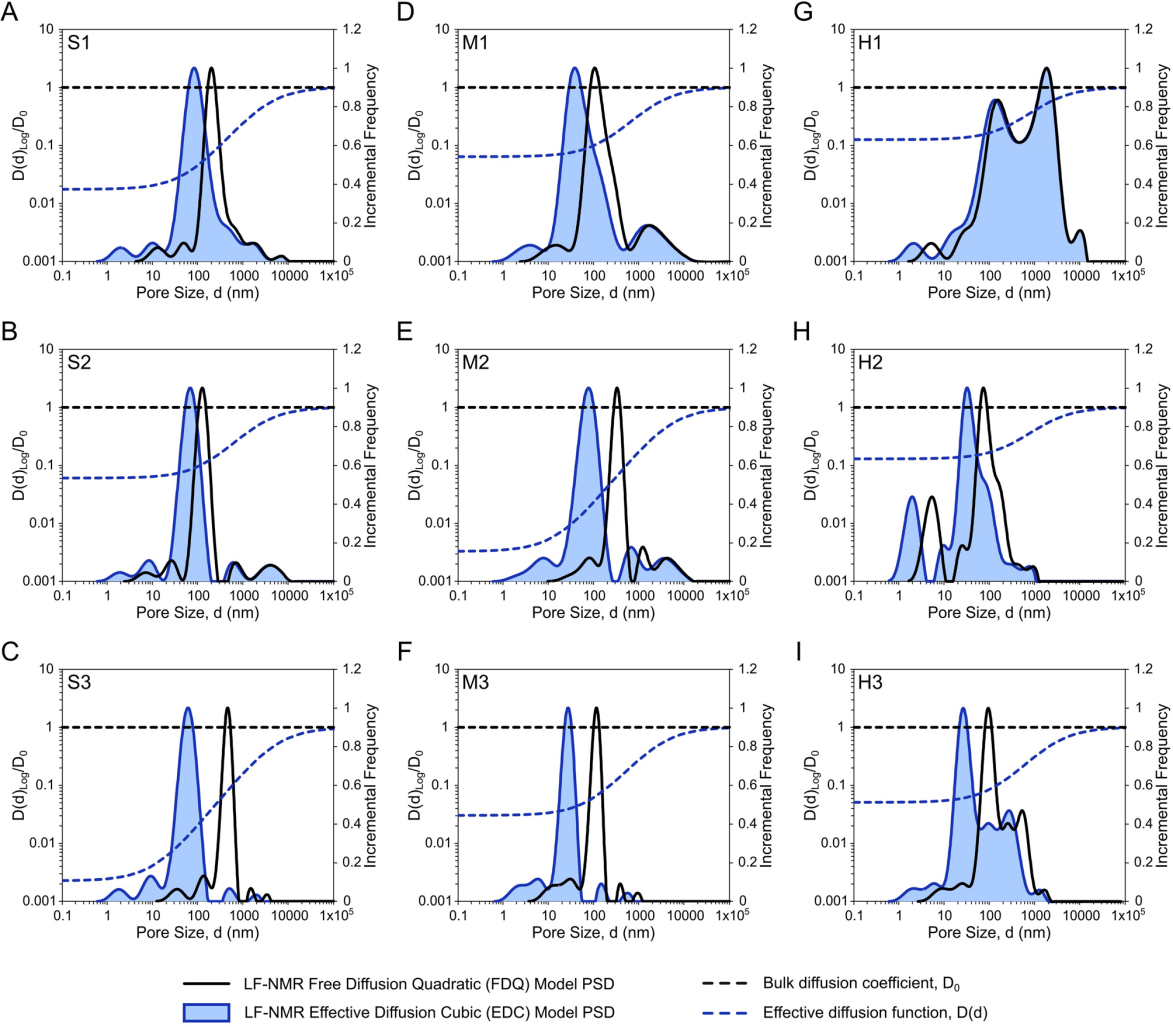
Results of EDC and FDQ models LF-NMR PSD estimation across representative rock-core samples. Comparison of different diffusion behavior assumptions on LF-NMR pore size distributions Estimation results obtained using the free diffusion quadratic (FDQ, black lines) and effective diffusion cubic (EDC, blue-filled areas) models across nine studied samples.**(A–C)** sandstones (S1-S3); **(D–F)** mudstones (M1-M3), and **(G–I)** heteroliths (H1-H3). Both models’ PSD results were derived assuming EDC *ρ*_*2*_ values and the same internal gradient functions, G(d), obtained using magnetic susceptibility data (Table 1). The left axis shows the magnitude of the normalised effective diffusion functions D(d)_Log_/D_0_ (dashed blue lines), obtained during PSD conversion parameters fitting and bulk diffusion *D*_*0*_ baseline (dashed black lines).

On the other hand, estimation of reliable PSDs was impossible using the Free Diffusion Quadratic (FDQ) model. The smallest T_2_-PSD conversion errors were obtained with not physically valid surface relaxivity, *ρ*_*2*_ *= 0*, except for sample H1 with the largest pore sizes of mode in range d > 1000 nm (Fig. 3G). Therefore, for all nine investigated samples, we used *ρ*_*2*_ values estimated using the EDC approach (Fig. 3). This has allowed us to evaluate solely the impact of effective diffusion function implementation on LF-NMR PSD conversion, as both EDC and FDQ models consider the same magnitude of internal gradients, *G(d)*. In all cases, the FDQ-derived PSDs displayed a bias toward larger pore sizes, particularly in the micropore domain (*d* < 100 nm). This was most evident in samples S1, S3, M2, and M3, with the highest magnetic susceptibilities (Table 1), where the FDQ approach yielded narrow peaks in the 100–500 nm range that were not supported by the already fitted to reference PSD data, EDC model distributions (Fig. 3C, E-F, I).

The strongest effect of applied effective diffusion functions, *D(d)*_*Log*_ is observed with highest PSD shifts in samples with EDC PSD peaks in regions below 100 nm (Fig. 3). In samples such as S3 and M2, where *D(d)*_*Log*_*/D*_*0*_ fell below 0.01 for pores under 10 nm, the EDC model results exhibited most effectively suppressed influence of the internal gradients, *G(d)* (Fig. 3C, E). Notably, in sample H1 with *D*_*∞*_ higher than the order of 10^− 12^ (Table 1) and mode in the range of *d* > 1000 nm, the difference between FDQ and EDC approaches narrowed (Fig. 3G). At the same time, for sample H2, with very similar *D*_*∞*_ (Table 1) but with a mode in the range of *d* < 100 nm, such narrowing was not observed (Fig. 3H).

**Table 1:** Calibration and surface relaxation parameters for T_2_-PSD conversion.

Lithology	Sample	χ_Sample_	NDL ρ_2_	EDC ρ_2_	D_∞_	d_c_	NDL T_2Slm_	EDC T_2Slm_	ΔT_2Slm_
		(-)	(µm/s)	(µm/s)	(m^2^/s)	(µm)	(ms)	(ms)	(ms)
Sandstone	S1	1.97 × 10^− 4^	1.00	0.83	1.56 × 10^− 12^	3.35	11.53	15.24	3.71
	S2	1.08 × 10^− 4^	0.73	0.62	5.31 × 10^− 12^	2.56	11.17	15.25	4.09
	S3	5.61 × 10^− 4^	0.75	0.20	1.98 × 10^− 13^	5.28	7.46	33.50	26.04
Mudstone	M1	1.07 × 10^− 4^	0.34	0.21	5.67 × 10^− 12^	2.52	19.05	39.33	20.27
	M2	4.48 × 10^− 4^	1.35	0.96	2.90 × 10^− 13^	4.85	8.85	13.28	4.42
	M3	1.68 × 10^− 4^	0.49	0.16	2.67 × 10^− 12^	2.98	4.63	18.91	14.28
Heterolith	H1	7.16 × 10^− 5^	1.41	1.39	1.10 × 10^− 11^	2.17	29.37	32.10	2.73
	H2	7.54 × 10^− 5^	0.30	0.12	1.14 × 10^− 11^	2.17	5.52	25.82	20.29
	H3	1.21 × 10^− 4^	0.37	0.11	4.50 × 10^− 12^	2.65	18.43	60.61	42.18

Measured volume magnetic susceptibility values (*χ*_*sample*_) were used to calculate *G(d)* input functions for free diffusion quadratic (FDQ) and effective diffusion cubic (EDC) calibration processes. Parameters *ρ*_*2*_ and *D*_*∞*_ were fitted during the calibration of the negligible diffusion linear (NDL) and EDC models to reference MICP and LTNA data. Surface relaxation log-means (T_2Slm_) and their difference (ΔT_2Slm_) were derived from the resulting NDL and EDC pore size distributions for the studied rock-core samples.

**Fig. 4:**
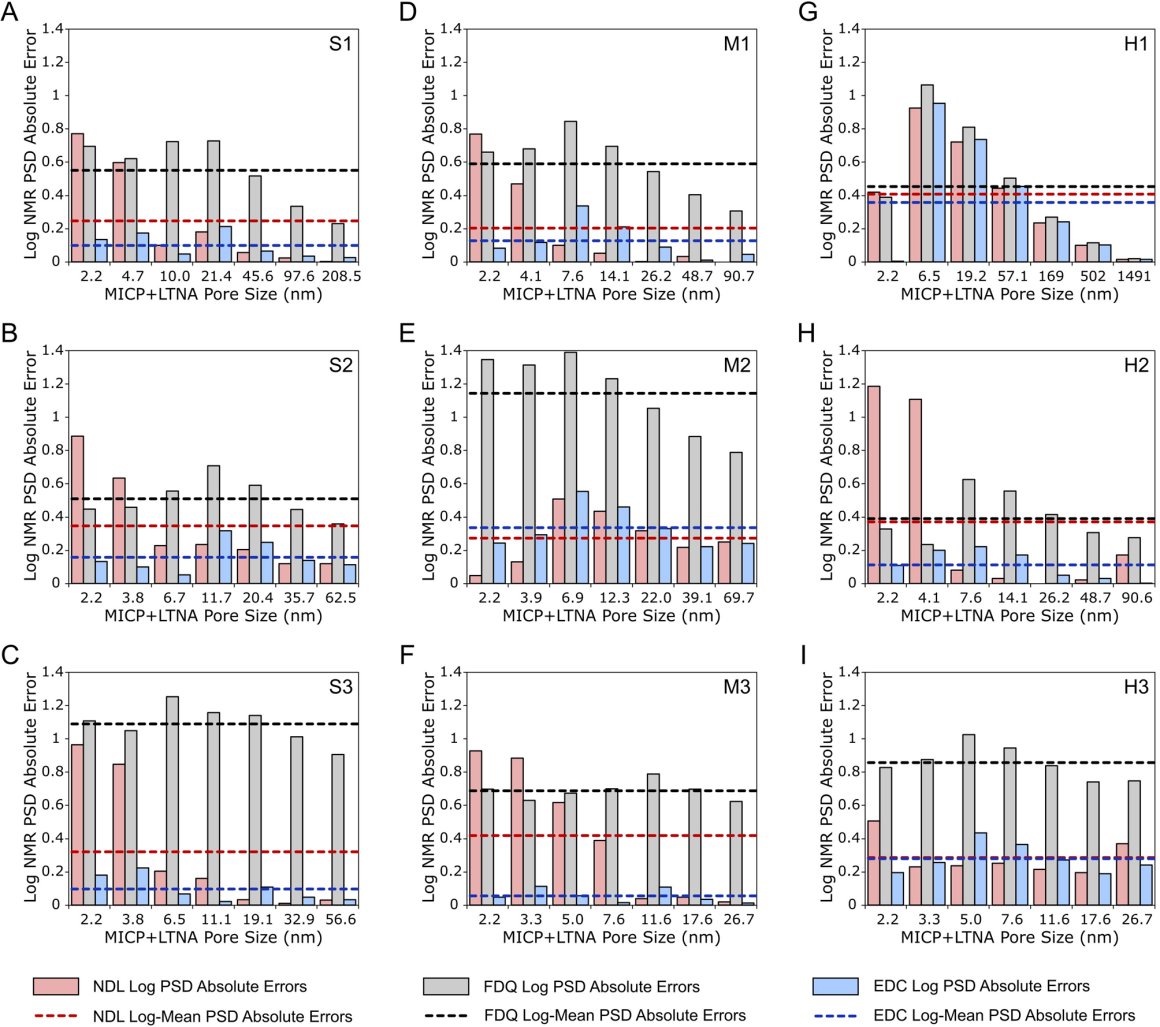
Results of error minimisation obtained during the tested T_2_-PSD conversion models calibration. Log PSD absolute fitting errors obtained during calibration of negligible diffusion linear (NDL, red bars), free diffusion quadratic (FDQ, grey bars) and effective diffusion cubic (EDC, blue bars) models to measured MICP + LTNA PSD data for **(A–C)** sandstones (S1-S3); **(D–F)** mudstones (M1-M3) and **(G–I)** heteroliths (H1-H3) for seven chosen conversion points fulfilling assumption of cumulative curves conformity. Horizontal dashed lines with corresponding colours indicate the log-mean PSD absolute fitting errors of each method.

For the quantitative evaluation of PSD estimation results, log PSD absolute fitting errors between LF-NMR-derived PSDs and the reference MICP + LTNA distributions for all analysed rock samples and models were summarised (Fig. 4). Across all but one case (Fig. 4E), the EDC model results yielded lower or substantially reduced log PSD mean absolute error values compared to FDQ and NDL models. In many instances, the reduction was more than an order of magnitude (Fig. 4C, F, H). Additionally, the EDC results demonstrated a more uniform distribution of errors than the other two models. The application of the FDQ model resulted in highly non-uniform error distributions, often showing sharp spikes in fitting errors for the micrometric pore sizes regime (between 5 and 10 nm). On the other hand, the NDL model, which neglects both internal gradients influence and restriction of diffusion effects on spin-spin relaxation, showed increase in fitting errors in almost all small pore size bins (*d* < 5 nm), apart from one sample, M2 (Fig. 4E). Although its error profile was less severe than FDQ in six out of nine tested samples (Fig. 4A-D, F-G).

### Analysis of derived surface relaxation times and their correlation with measured PSD

**Fig. 5:**
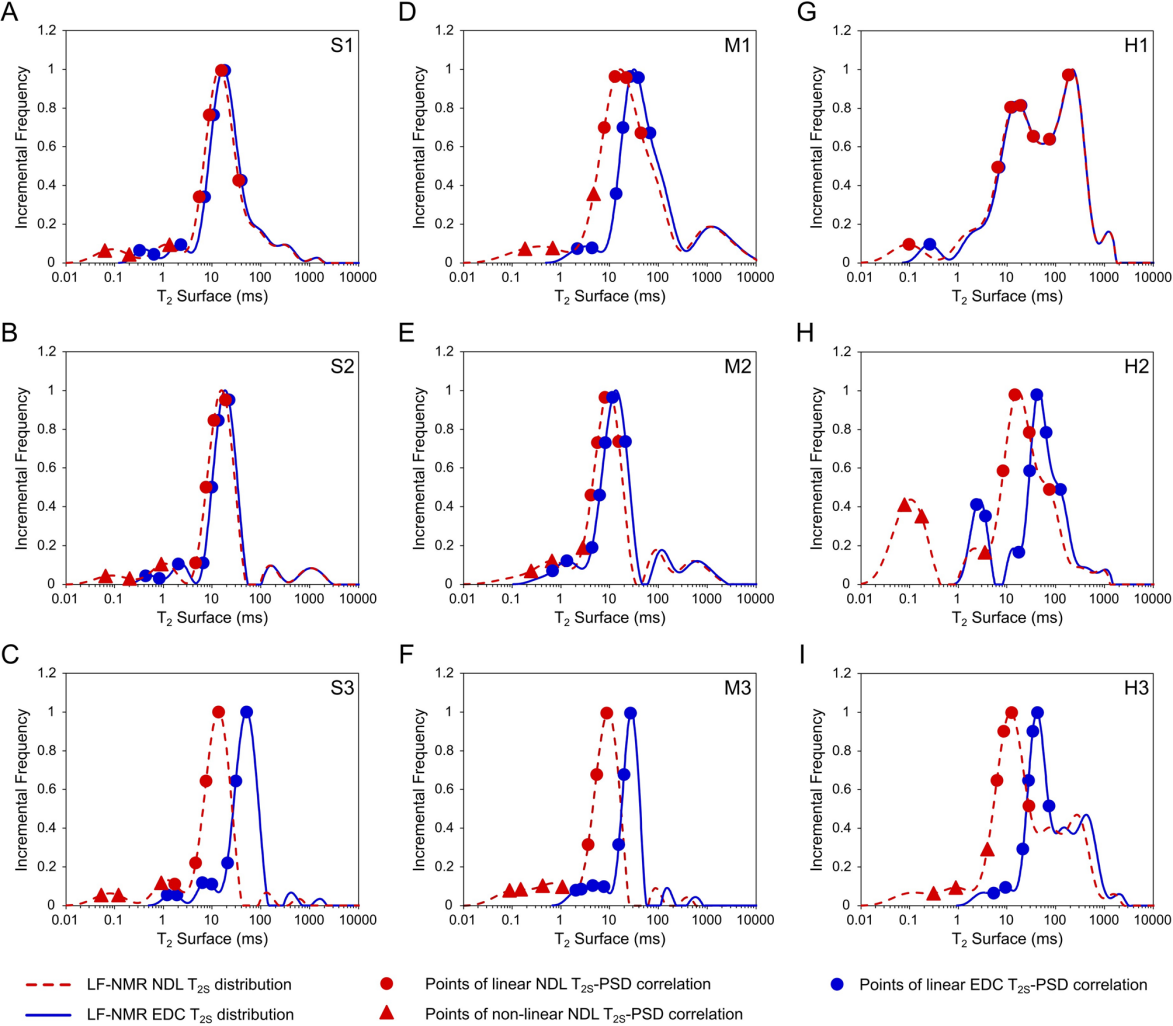
Surface relaxation distributions obtained during T_2_-PSD conversion using NDL and EDC models. Surface relaxation data estimated for nine tested rock-core samples **(A-C)** sandstones (S1-S3); **(D-F)** mudstones (M1-M3) and **(G-I)** heteroliths (H1-H3) using Negligible Diffusion Linear (NDL, red dashed lines) and Effective Diffusion Cubic (EDC, blue solid lines) models. Red and blue markers denote conversion points used later during LF-NMR T_2S_-PSD correlation, indicating points of linear (circles) and non-linear (triangles) regression trends observed between estimated T_2S_ and corresponding, measured by reference MICP and LTNA methods, PSD values (Fig. 6).

To further validate the quality of the LF-NMR pore size distribution estimation using NDL and EDC models, we investigated the extent of diffusion component (*T*_*2D*_) influence on the surface relaxation times (*T*_*2S*_) estimation from PSD data. The FDQ method, in which it was impossible to estimate the surface relaxivity, *ρ*_*2*_, was excluded from this analysis. Applying the NDL model, in which T₂ relaxation is assumed to be attributed entirely to surface and bulk relaxation, resulted in significantly lower surface relaxation times then these obtained using EDC (Fig. 5). The shift towards longer times of EDC results was most pronounced in samples with the lowest surface relaxivity, such as S3, M3, and H2-3 (Table 1), where the contribution of surface relaxation in spin-spin relaxation was the most constrained. In such cases, the application of the NDL model introduced the most visible *T*_*2S*_ distribution broadening to short-time regions (Fig. 5C, F, H-I).

**Fig. 6:**
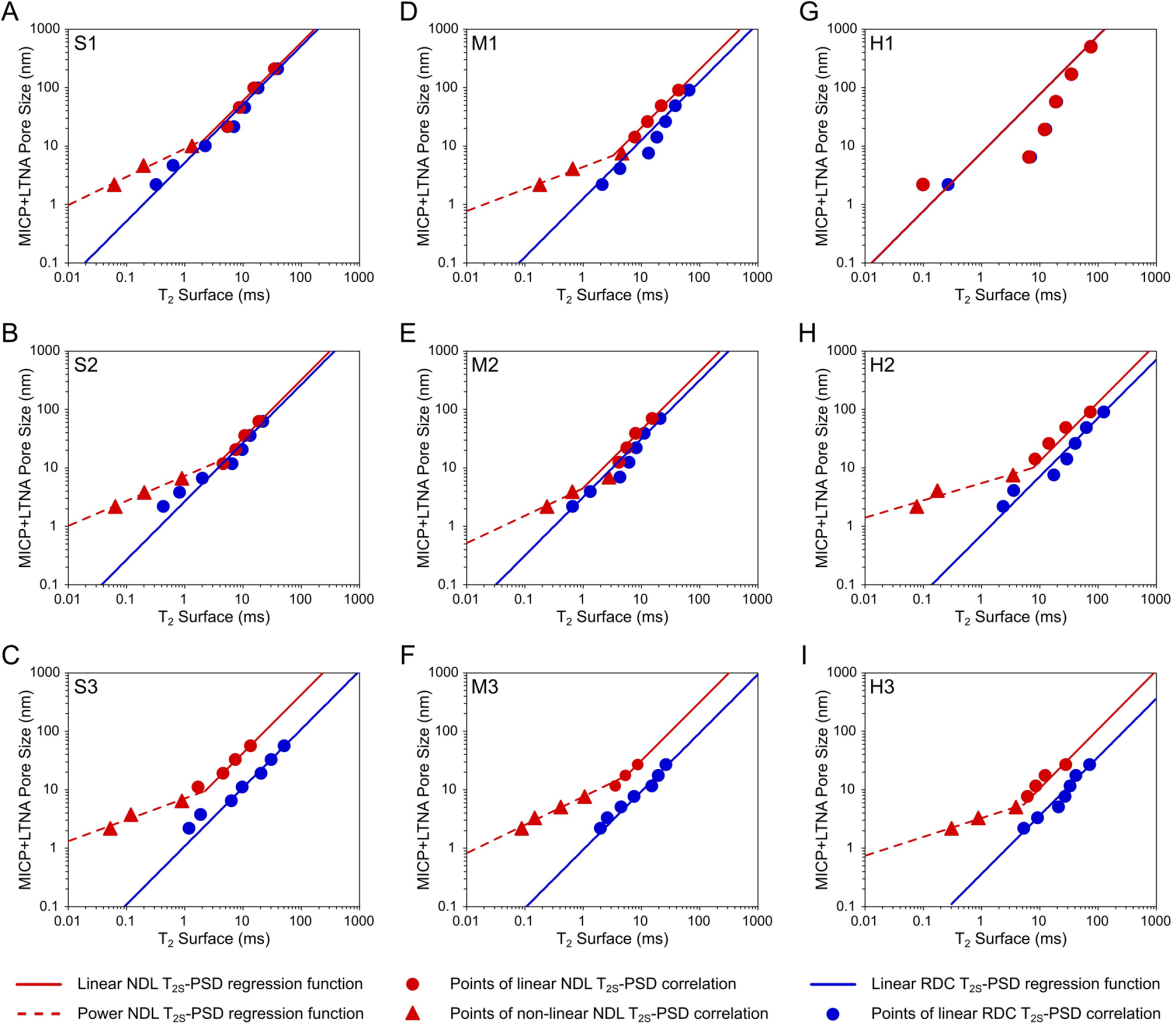
Regression of obtained surface relaxation times (*T*_*2S*_) versus measured MICP + LTNA pore sizes. T_2S_ values of conversion points chosen during PSD Estimation workflow, obtained using NDL (red markers) and EDC (blue markers) models for **(A-C)** sandstones (S1-S3); **(D-F)** mudstones (M1-M3); **(G-I)** heteroliths (H1-H3). Solid lines with corresponding colours represent the linear regression trends across NDL (red circles) and EDC (blue circles) data points. Dashed lines highlight the non-linear fit of NDL data (red triangles) in low surface relaxation time segments. Positions of analysed conversion are marked on the obtained surface relaxation times distributions (Fig. 5).

Additionally, the application of the EDC method in *T*_*2S*_ estimation not only mitigated this artificial broadening but also improved the continuity of the linear correlation between surface relaxation and pore size (Fig. 6). EDC T_2S_ values followed a more coherent, physically consistent linear trend with measured PSD values across all samples, apart from H1 (Fig. 6G). At the same time, the NDL *T*_*2S*_ values deviated from this trend, particularly at the low ends of the T_2S_ range. For instance, samples such as S3, M3, and H2-3 showed the strongest divergence between the conventional, NDL, and introduced EDC model. In these cases, surface relaxation times of conversion points (Fig. 6C, F, H-I) most visibly failed to match the physically consistent, linear log-log scaling nature of the T_2S_-PSD relation, while the regression lines based on EDC T_2S_ values provided improved linearity throughout the entire distributions. In contrast, sample H1 with a wider distribution of larger pores of mode in range d > 1000 nm (Fig. 2G), was characterized by a comparably weak linear T_2S_-PSD correlation in the case of all analysed points and models used (Fig. 6G).

### Evaluation of pore space characteristics obtained using the EDC model T_2_-PSD conversion

**Fig. 7:**
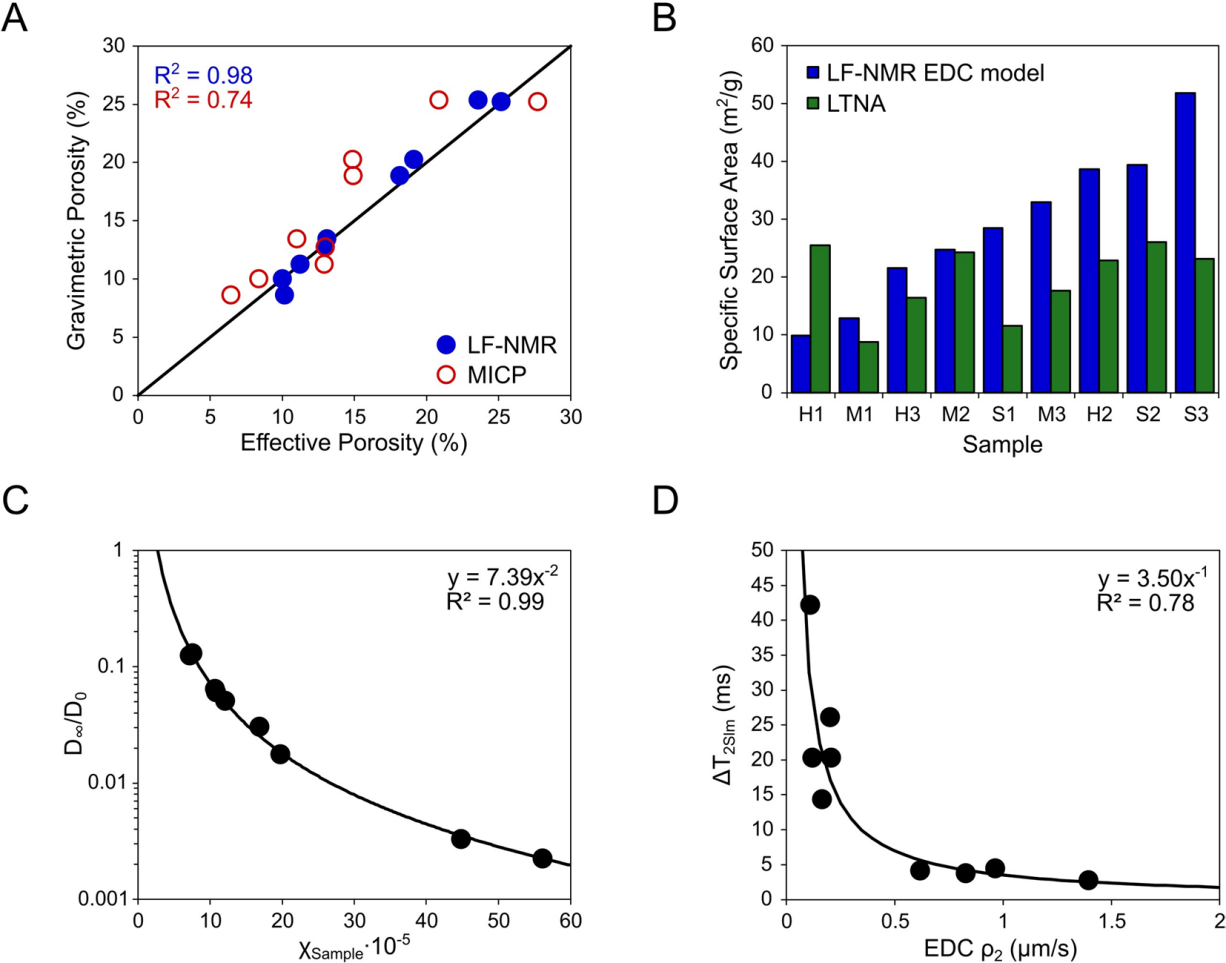
Validation of porosity and surface area derived using the EDC model and investigation of magnetic susceptibility and surface relaxivity effects on T_2_-PSD conversion. **(A)** Comparison of effective porosity obtained by differential LF-NMR, MICP, and gravimetric methods; **(B)** Comparison of specific surface area measured using LTNA and estimated using LF-NMR EDC model PSD conversion results; **(C)** Inverse correlation between sample magnetic susceptibility, *χ*_*Sample*_, and normalised restricted diffusion coefficient, *D*_*∞*_*/D*_*0*_; **(D)** Inverse correlation between surface relaxivity fitted from EDC model (*EDC ρ*_*2*_) and the shift in log-mean surface relaxation time (ΔT_2Slm_) between EDC and NDL models.

To evaluate the broader implications of effectively correcting the diffusion component, *T*_*2D*_ influence on LF-NMR pore space characterization, we analysed the relationship between measured volume magnetic susceptibility and that obtained during EDC model calibration, restricted diffusion, *D*_*∞*_, and surface relaxivity parameters, *p*_*2*_. Furthermore, we validate porosity, PSD, and specific surface area results determined using the EDC model with complementary, LTNA, MICP, and gravimetric porosity measurements (Fig. 7; Table 2).

**Table 2:** Comparison of petrophysical parameters derived using LF-NMR EDC model T_2_-PSD conversion and reference methods for all investigated rock-core samples.

Parameter		PSD log-mean (nm)		Specific surface area (m^2^/g)		Effective porosity (%)			K (mD)
Lithology	Sample	MICP + LTNA	LF-NMR (EDC)	LTNA (BET)	LF-NMR (EDC)	MICP	Gravimetric	LF-NMR (Differential)	MICP
Sandstone	S1	22.4	75.6	11.61	28.46	14.9	18.8	18.2	4.95 × 10^− 2^
	S2	15.4	56.5	26.04	39.37	27.7	25.2	25.2	2.80 × 10^− 3^
	S3	16.1	40.2	23.20	51.84	20.9	25.4	23.6	2.20 × 10^− 3^
Mudstone	M1	17.0	48.4	8.79	12.84	8.4	10.0	10.0	2.80 × 10^− 3^
	M2	15.3	76.7	24.25	24.75	14.9	20.3	19.1	2.50 × 10^− 3^
	M3	10.8	18.5	17.67	32.92	13.0	12.7	13.0	3.00 × 10^− 4^
Heterolith	H1	49.6	268.6	25.50	9.89	12.9	11.2	11.2	5.07 × 10^− 1^
	H2	13.8	18.6	22.84	38.66	6.5	8.6	10.2	5.00 × 10^− 4^
	H3	10.0	39.3	16.47	21.58	11.0	13.4	13.1	2.00 × 10^− 4^

LF-NMR effective diffusion cubic (EDC) model specific surface areas were calculated assuming spherical pore shapes. LF-NMR effective porosity was obtained from differential distributions derived by Subtracting the signal of the dry sample from the 100% kerosene saturated sample before performing the inverse Laplace transformation (ILT). Differential distributions also provided the base LF-NMR input for T_2_-PSD conversion.

Across all nine samples, the log-mean values obtained from EDC model LF-NMR PSD conversion results were consistently larger, often by an order of magnitude, in comparison to those obtained from combined MICP + LTNA distributions. The difference was particularly pronounced in samples with higher permeability, such as S1 and especially H1, where NMR yielded a value of 268.6 nm compared to just 49.6 nm from reference methods. Conversely, the lowest EDC-derived PSD log-mean values (e.g., M3 and H2) still exceeded their MICP + LTNA counterparts by factors of 1.5–2 even in ultra-low permeability systems (Table 2). In addition, the LF-NMR effective porosities exhibited excellent agreement with gravimetric data (R^2^ = 0.98), while the MICP results showed increased scatter (R^2^ = 0.74) around the x = y regression line (Fig. 7A). Moreover, the LF-NMR specific surface areas, obtained using the EDC PSD model, exceeded LTNA-measured values in almost all cases (besides sample H1), often by a factor of 2–3 (Fig. 7B).

In terms of relaxometric parameters, a strong inverse relationship (R^2^ = 0.99) was observed between the normalised restricted diffusion coefficient, *D*_*∞*_/D_0,_ and the bulk magnetic susceptibility, *χ*_*Sample*_, in investigated rock-core samples (Fig. 7C). Additionally, an absolute shift in log-mean surface relaxation time (*ΔT*_*2Slm*_) between NDL and EDC model results was observed to be inversely correlated with the surface relaxivity estimated accounting for diffusion component influence, EDC *ρ*_*2*_. In this correlation, the largest *ΔT*_*2Slm*_ shifts (20–40 ms) could clearly be associated with the lowest (< 0.5 μm/s) *ρ*_*2*_ values (Fig. 7D).

## Discussion

Our work attempted to bridge the gap in the accurate pore size distributions modelling and practical LF-NMR petrophysical analysis by presenting an experimentally validated methodology. Employed EDC approach enables the simultaneous estimation of surface relaxivity, governing the surface relaxation and the effective diffusion function, *D(d)*, which, alongside the induced internal magnetic field gradients, *G(d)*, determines the diffusion component contribution. Simplified estimation methods, as applied in previous studies^4,9,26,27,31,32^which introduce a non-linear relationship between T_2_ times and pore sizes, seem to offer only a coarse approximation for diffusion and internal gradient effects correction. Such approaches should be applied cautiously and be limited to the low-end parts of the T_2_ distribution that deviate from the linear T_2S_–PSD correlation trends expected under surface relaxation-dominated, fast diffusion regime conditions.

In low-field NMR theory, surface relaxation should enable a direct linear regression with a shift equal to 0 between *T*_2S_ and pore diameter, *d*, via transverse surface relaxivity (*ρ*_2_) and pore surface-to-volume (*S/V*) factors^19,20^. Nevertheless, our results show that in pores below ~ 100 nm, the T₂ signal reflects surface and diffusion-related mechanisms, both of which are pore-size dependent. As shown in earlier studies, the dependence of surface relaxation time on pore size is obvious. However, classical considerations about diffusion assume that the magnitude of its restriction depends only on the tortuosity^39–41,47^. However, we found that rock magnetic susceptibility also governs effective diffusion, with higher *χ*_Sample_ producing stronger *D*_*∞*_ suppression. Moreover, the effective diffusion influence on T_2_ relaxation in nano- and micropores scales with surface relaxivity: samples with ρ_2_ < 0.5 μm/s exhibited the largest diffusion contribution to overall relaxation.

Failure to account for restricted diffusion and internal gradients induced by susceptibility gradients, as in the Negligible Diffusion Linear (NDL) model, leads to systematic underestimation of NMR-derived PSDs, sometimes producing non-physical values. The Free Diffusion Quadratic (FDQ) model introduces a gradient correction through *G(d)*, but neglects effective diffusion suppression, resulting in overestimation of surface relaxivity, *ρ*_2_​. These limitations highlight the inadequacy of assuming a constant bulk diffusion coefficient in nano- and microporous media. In contrast, the Effective Diffusion Cubic (EDC) framework introduces a pore-size-dependent effective diffusion coefficient, *D(d)*, expressed by a logistic function that faithfully approximates the Padé form. This representation captures the suppression of molecular diffusion in restricted environments and accurately quantifies its impact on spin–spin relaxation. As a result, the EDC model provides a consistent formulation of the diffusion-related contribution, *T*_2D_​, to the overall relaxation process, thereby improving the accuracy of surface relaxation estimation (*T*_2S_​) and ensuring physically realistic conversion of T_2_ ​distributions into PSDs.

By applying the proposed EDC model, key limitations of the conventionally used NDL and FDQ models for T_2_-PSD transformation could be overcome, leading to: (1) improved alignment between NMR-derived PSDs and those obtained by MICP and LTNA methods; (2) reduced conversion errors and the elimination of non-physical artefacts in the sub-nanometric and nanometric range; (3) physically valid linearity of the T_2S_-PSD correlation in the full range of relaxation times and pore sizes. Notably, the proposed EDC model showed the most stable performance across all lithologies and pore size ranges with uniformly minimised local fitting errors. Its low mean fitting error values and flatter error distributions highlight the robustness of incorporating pore-size-dependent effective diffusion function in PSD estimation.

In addition, the restricted diffusion coefficients, *D*_∞_ obtained during the EDC model calibration ranged in the order of 10⁻¹¹ to 10⁻¹³ m²/s, which aligns with effective diffusion values reported in independent diffusion measurements of iodide and HTO tracers^48^, water vapor^49^, diiodomethane^50^ and methane^51^ in the tight pore networks of shale formations. This agreement supports the validity of the EDC model in capturing effective diffusion coefficients solely through PSD fitting, without requiring dedicated diffusion experiments. Furthermore, EDC-derived PSDs yielded higher specific surface areas than LTNA and stronger agreement with gravimetric porosity than MICP. These results demonstrate the potential of the LF-NMR EDC approach to surpass the limitations of LTNA and MICP, whose applicability in tight systems is restricted by connectivity thresholds and destructive sample preparation.

Despite the advances achieved, several limitations remain. The EDC model relies on spherical pore geometry, single-fluid saturation, and uniform wetting, assumptions that may not hold in shales, fractured rocks, or carbonates with complex surface chemistry. In particular, adopting a constant spherical shape factor (F_s_ = 3) provides internal, methodological consistency but represents a strong simplification for nano- and microporous domains, where slit-like or irregular geometries dominate and may bias surface area and ρ_2_ estimations. In addition, the percolation-based framework used to establish the T_2_-PSD conversion framework performed less reliably in sample H1 with a dominant pore population in the range of d > 1000 nm, consistent with earlier reports of its limited applicability in rocks with broad pore size distributions^9,43,44^.

Another limitation is that the estimation of the restricted diffusion, *D*_*∞*_, crucial in obtaining effective diffusion coefficients, *D(d)*, remains model based. As can be seen in the simulation results, both the structural function parameters, proportionality factor of *θ* from the Padé approximation, and the *d*_*c*_ from the logistic model, exhibit exponential correlation with the normalised restricted diffusion coefficient, *D*_*∞*_/*D*_*0*_. However, the logistic approximation lost accuracy below *D*_*∞*_/*D*_*0*_ = 0.1 threshold. Nevertheless, according to our results, even for highly confined pore systems of *D*_*∞*_ *= 8.8 × 10*^*− 13*^, the choice of logistic model for effective diffusion approximation will affect the *D(d)* estimates by less than 1.4 times in comparison to Padé approximation results.

## Conclusions

The results of this study demonstrate that neglecting restricted diffusion and susceptibility-induced internal gradients leads to systematic biases in LF-NMR-derived pore size distributions (PSDs) for rocks with nano- and micropores. Conventional models, such as NDL and FDQ, either underestimate or overestimate PSDs by assuming bulk-like diffusion behavior, resulting in non-physical artefacts and unreliable surface relaxivity estimates.

By introducing a pore-size-dependent effective diffusion coefficient, D(d), represented through a logistic function faithfully approximating the Padé form, the Effective Diffusion Cubic (EDC) model provides a rigorous framework for capturing diffusion suppression effects. This approach enables accurate separation of surface- and diffusion-related contributions to T_2_ relaxation, yielding physically consistent PSDs across nano- and microporous domains. The EDC model not only enhances the alignment of LF-NMR results with independent reference techniques, such as MICP and LTNA, but also provides more accurate estimates of specific surface area and porosity. Its robustness across diverse lithologies and pore size ranges confirms its applicability as a reliable tool for petrophysical evaluation of tight formations.

Future work should integrate direct NMR diffusion experiments and extend the framework to 2D and 3D approaches (D-T_2_, DWI, DTI) to refine the interpretation of diffusion dynamics in nanoporous and microporous spaces. Validation across diverse lithologies, including shales and multi-fluid rock pore systems, will be essential to demonstrate the robustness of the method. Importantly, the EDC framework is in principle applicable to downhole NMR logging, provided that formation-specific calibration is available. Further studies linking core-based calibration with logging conditions will therefore be a key step toward transferring this methodology from laboratory experiments to well-log interpretation.

## Supplementary Information

Below is the link to the electronic supplementary material.

Supplementary Material 1

## Data Availability

All data relevant to this study are available in the main text or the supplementary materials. The XLSX data that support the findings of this study and the XLSX script used in effective diffusion simulations are available in the Mendeley Data repository with the identifier 10.17632/7rd72pdh2x. Additional information is available from the corresponding author upon request.
