# Supplementary material for: Modelling effective diffusion for accurate NMR pore size analysis in nano- and microporous rocks
Source: Sci Rep. 2025 Oct 21;15:36688. doi: 10.1038/s41598-025-20379-x (PMC12540705; doi:10.1038/s41598-025-20379-x)
Supplement: Supplementary file 1 — Supplementary Material 1 [file 41598_2025_20379_MOESM1_ESM.docx]

Supplementary Materials for

**Modelling effective diffusion for accurate NMR pore size analysis in nano- and microporous rocks**

Michał Fajt,^1^* Grzegorz Machowski,^1^ Bartosz Puzio,^1^ Artur T. Krzyżak^1^

^1^AGH University of Krakow, Faculty of Geology, Geophysics and Environmental Protection,
al. Adama Mickiewicza 30, 30-059 Krakow, Poland.

*Corresponding author e-mail: [mfajt@agh.edu.pl](mailto:mfajt@agh.edu.pl)

**This PDF file includes:**

Figs. S1 to S2

References (1)


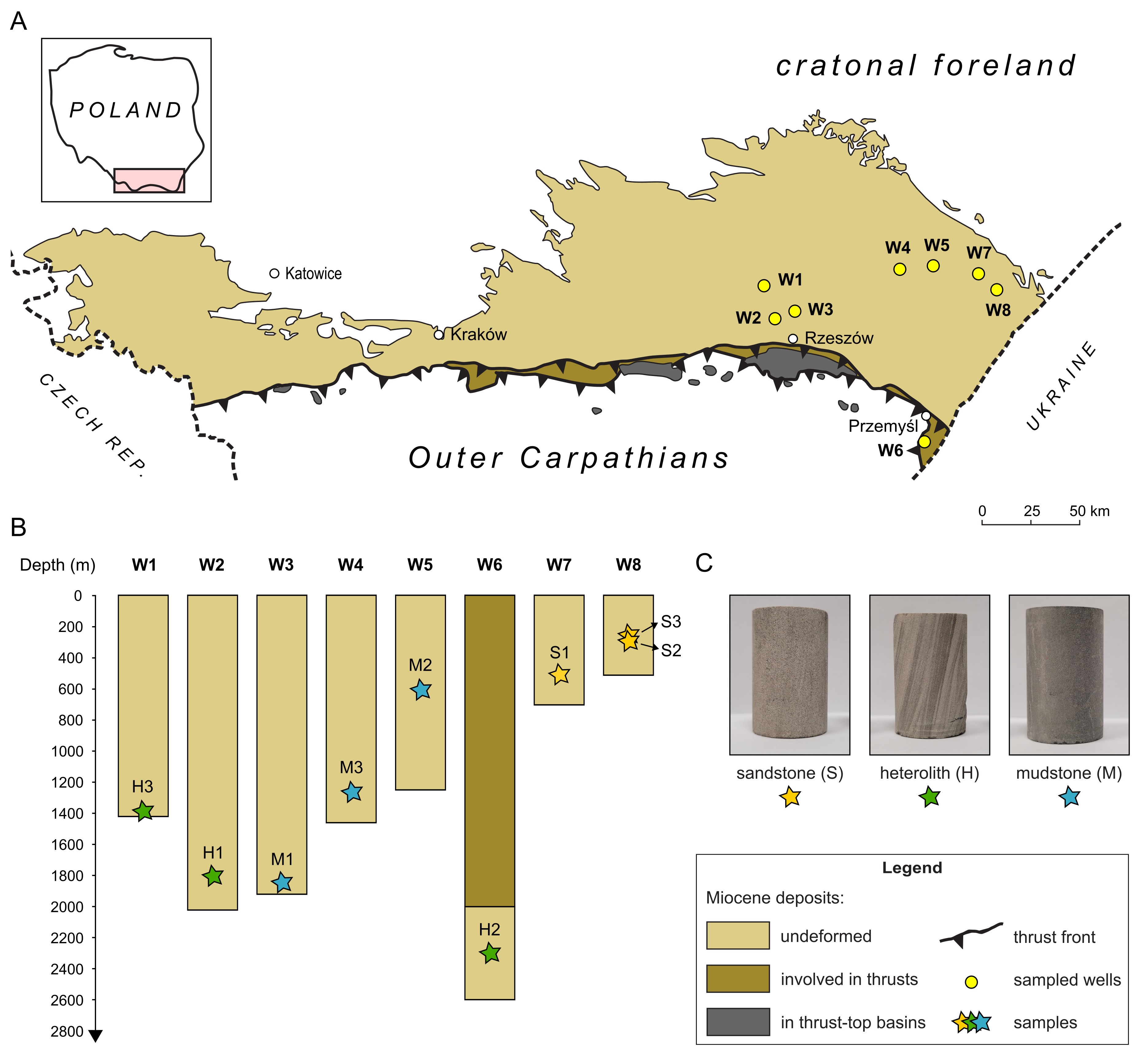


Fig. S1. Sample localisation and geological context of the studied wells in the Polish Carpathian Foredeep

**(A)** Map of study area representing the extent of Miocene sediments after Porębski and Warchoł,^1^ with an indication of the sampled wells W1 – W8); **(B)** Depth profiles of the Miocene sediments in sampled wells with sampling intervals indication; **(C)** Representative core samples of the three main studied lithotypes: sandstone (S), heterolith (H), and mudstone (M).


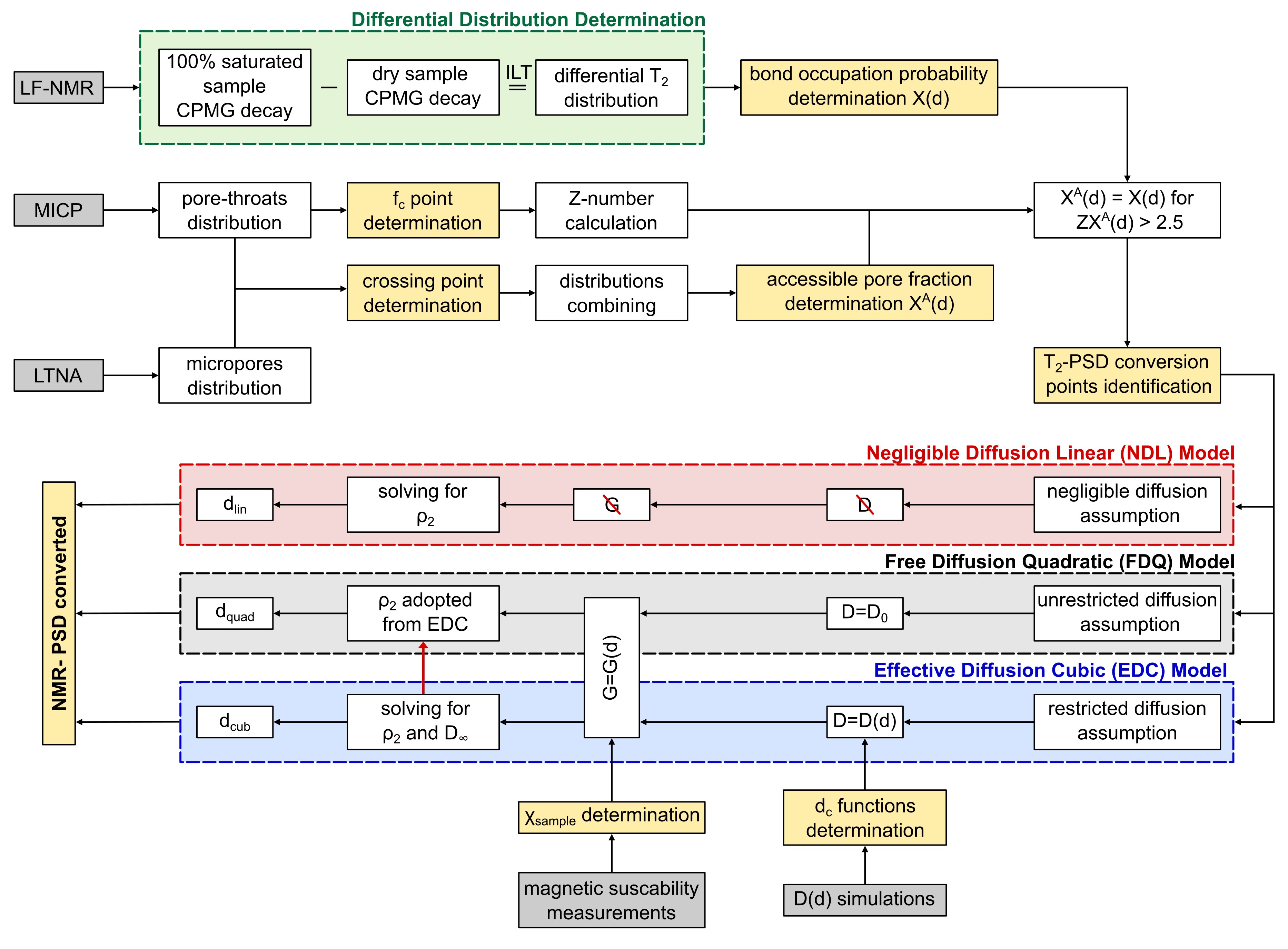


Fig. S2. Workflow for pore size distribution (PSD) determination using integrated LF-NMR, MICP, and LTNA data.

Flowchart outlining the data integration and modelling process used to convert NMR relaxation distributions into absolute pore size distributions (PSD), including input data from low-field NMR, mercury intrusion porosimetry (MICP), and low-temperature nitrogen adsorption (LTNA). Three T_2_-PSD conversion models – Negligible Diffusion Linear (NDL, red), Free Diffusion Quadratic (FDQ, light grey), and Effective Diffusion Cubic (EDC, blue) were employed to evaluate the role of different diffusion assumptions on PSD estimation results. The process of obtaining LF-NMR differential distributions is shown in green. Measurement and simulation methods used are depicted in dark gray. Main data processing steps are highlighted in yellow.

References

1. Porębski, S. J. & Warchoł, M. Znaczenie przepływów hiperpyknalnych i klinoform deltowych dla interpretacji sedymentologicznych formacji z Machowa (miocen zapadliska przedkarpackiego). *Przegląd Geologiczny* **54**, 421–429 (2006).
